# Distribution of Type I Restriction–Modification Systems in *Streptococcus suis*: An Outlook

**DOI:** 10.3390/pathogens5040062

**Published:** 2016-11-18

**Authors:** Niels Willemse, Constance Schultsz

**Affiliations:** 1 Department of Medical Microbiology, Academic Medical Center, University of Amsterdam, Meibergdreef 9, 1105 AZ Amsterdam, The Netherlands; schultsz@gmail.com; 2 Department of Global Health-Amsterdam Institute for Global Health and Development, Academic Medical Center, University of Amsterdam, Pietersbergweg 17, 1105 BM Amsterdam, The Netherlands

**Keywords:** *Streptococcus suis*, prophage, Type I restriction–modification system, pfam, genomic analysis, DNA inversions

## Abstract

*Streptococcus suis* is a porcine commensal and pathogen with zoonotic potential. We recently identified a novel Type I restriction–modification (R–M) system in a zoonotic *S. suis* clone which has emerged in the Netherlands. Here, we describe the DNA inversions in the specificity subunit of this system in *S. suis* serotype 2, clonal complex 20 and explain the absence of domain movement by the absence of repeats. In addition, we identified a core Type I R–M system present in 95% of the isolates and found an association of the distribution of Type I R–M systems in the *S. suis* genome with population structure. We speculate on the potential role of Type I R–M systems in *S. suis* given the recently described associations of Type I R–M systems with virulence and propose future research directions.

## 1. Introduction

Restriction–modification (R–M) systems are protein complexes which protect the host bacterium from invasion by foreign DNA through global methylation by methyltransferase (MTase) activity and digestion of the invaded DNA by restriction endonuclease (REase) activity [1]. However, the range of functions that R–M systems may have is expanding and includes stabilizing mobile genetic elements (MGEs) and gene regulation, potentially providing evolutionary fitness advantages and virulence under certain conditions [2]. The protection against foreign DNA may merely be a coincidental benefit of these functions [3].

**Figure 1:**
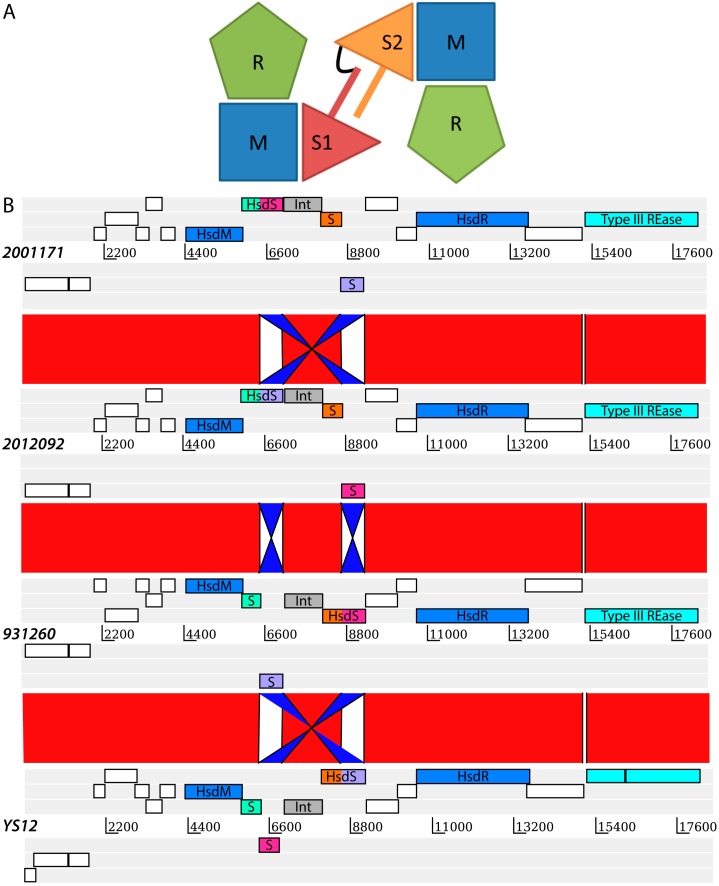
(**A**) Schematic representation of a Type I R–M system inspired by Loenen et al. [6]. A typical Type I R–M system is a pentameric protein complex consisting of two R subunits, two M subunits and an S subunit encoded by *hsdR*, *hsdM* and *hsdS* genes, respectively. The S subunit consists of two target recognition domains (TRDs), each of which can recognize a different DNA sequence. The two TRDs of the S subunit here are indicated by S1 and S2, separated by a bar which represents a separating amino acids sequence. The two TRD domains are covalently linked as illustrated with a black linker sequence; (**B**) Alignment of the prophage loci of four representative isolates containing four variants of the SsuCC20P Type I R–M system. DNA sequences are aligned using the Artemis Comparison Tool [18] which illustrates the DNA inversions that have taken place and resulted in four different *hsdS* genes. A complete *hsdS* gene consists of two Methylase_S (pfam01420) target recognition domains (TRDs), which are colored in the figure to illustrate the rearrangements. The putative four complete S subunits for each of the isolates are respectively; i: green/pink in isolate 2001171, ii: green/purple in isolate 2012092, iii: orange/pink in isolate 931260 and iv: orange/purple in isolate YS12. The two genes indicated in blue are the *hsdM* and the *hsdR* gene, respectively. The gene highlighted in cyan is a Type III restriction endonuclease. An integrase (tyrosine recombinase), which likely facilitates the demonstrated DNA inversions is indicated in grey. The complete annotation from 5′ to 3′ is presented in Supplementary Table S1.

Amongst the R–M systems, the Type I R–M systems in particular have been shown to be important for gene regulation and possibly pathogen virulence [4,5]. Three host specificity determinant (*hsd*) genes (*hsdS*, *hsdM* and *hsdR*), coding for a specificity (S), a modification (M) and a restriction (R) subunit, are necessary for a complete Type I R–M system (Figure 1A). A typical Type I R–M system consists of the pentameric protein complex 2R+2M+S. The trimeric protein complex 2M+S can act independently as a MTase [6] but the addition of two R subunits is required for REase activity. Each S subunit consists of two target recognition domains (TRD), each of which can bind to different specific sequences in the DNA. Each individual TRD can be exchanged, resulting in different TRD combinations, allowing for a great variety of R–M specificities. Both the MTase complexes as well as the REase complex make use of the same S subunit, which creates an efficient system for switching the R–M specificity of the host cell. The different combinations of TRDs in S subunits result in different global methylation patterns of the host DNA, which in turn may have an impact on global gene expression. Recently, this switching of S subunits in the Type I R–M system SpnD39III was shown to be linked to a switch from a carriage state to a virulent state in *Streptococcus pneumoniae* [5].

*S. suis* is a Gram-positive bacterium which is a major problem to pig farming, as well as a zoonotic pathogen causing meningitis and septicaemia [7,8]. *S. suis* is carried asymptomatically by up to 100% of pigs, but can become an opportunistic pathogen. The mechanisms responsible for switching from a carriage state to a virulent state are not well understood. Recently, we identified a novel Type I R–M system on a prophage in a zoonotic *Streptococcus suis* clone in the Netherlands [9]. Little is known about R–M systems in *S. suis* except for the Type II R–M system SsuDat1I which was described by Sekizaki et al. [10,11,12]. The Restriction Enzyme Database (REBASE) lists putative R–M systems which have been detected from whole genome sequencing data, but the large majority does not have (predicted) recognition sites or have not been characterized [13]. Here, we provide a detailed descriptive analysis of this novel Type I R–M system in *S. suis*. In addition, we describe the distribution of Type I R–M systems in *S. suis* and speculate on the origin and importance of R–M systems in *S. suis*.

## 2. Results and Discussion

We sequenced 116 *S. suis* isolates from the Netherlands and performed a whole genome analysis to search for genetic factors important for zoonotic potential [9]. The single *S. suis* serotype causing zoonotic infection in the Netherlands is serotype 2, which belongs to either multi-locus sequence type (MLST) clonal complex (CC) 1 or CC20 [14]. In contrast, serotype 9, belonging to CC16, is responsible for most porcine infections and is thought to be the most prevalent serotype in the pig population. In our genomic analysis, we focused on a comparison between these three major clonal complexes (CC1, CC16 and CC20) and found a prophage region in the CC20 isolates which was inserted in the genome between an integrase and the ribosomal proteins rpsI and rplM. This prophage contains a type I R–M system. Because the exact gene location of this system is unknown due to the draft nature of the genome sequences of the CC20 isolates, we cannot follow the REBASE systematic nomenclature approach for the novel Type I R–M system. Thus, for now, we unconventionally name the novel Type I R–M system SsuCC20P.

The sequences of all of the four prophage regions detected are illustrated in Figure 1B. The 18 kb prophage region consists of two transposons, an integrase, a Type III restriction endonuclease and all three subunits of a Type I R–M system (Supplementary Table S1) and appears similar to a prophage (remnant) example as described by Croucher et al. [15]. The M-subunit and the R-subunit showed 100% amino acid sequence identity with 100% coverage to a DNA methyltransferase (WP_024405918) and a deoxyribonuclease HsdR (WP_024405925) respectively, in a BLASTp search against the Genbank database. In contrast, we identified three variants of the S subunit of the Type I R–M system in the CC20 isolates and an additional fourth variant in the Genbank database which was present only in a single isolate YS12, from China. None of the three S subunits in the CC20 isolates resulted in high coverage and high identity BLAST hits against the Genbank database. Closer examination of the YS12 isolate, which had high identity and coverage with the M-subunit and R-subunit of CC20 isolates of the Netherlands, resulted in the discovery of a fourth (expected) variant of the S subunit. Figure 1B illustrates the DNA inversions that have occurred among the *hsdS* genes resulting in four *hsdS* variants. These DNA inversions are most likely facilitated by a recombinase which is located among the *hsdS* genes, and is a member of the same tyrosine recombinase family as CreX in *S. pneumoniae* [5,15]. These inversions do not appear to be specific to one lineage within CC20 (Supplementary Table S2).

We located SsuCC20P on a prophage region, which is considered a rare site on which to find R–M systems [16]. Prophages are a means of horizontal gene transfer between bacterial isolates which may result in fast diversification of bacterial species, such as *S. pneumoniae* [15]. The prophage region, which was found on single contigs in all CC20 isolates, was also present in its entirety in the YS12 isolate. The YS12 isolate is a sequence type (ST) 17 isolate that belongs to CC20. In contrast to ST17 isolates from the Netherlands, which express serotype 4 capsular polysaccharides, YS12 expresses serotype 7. To our knowledge, this is the only *S. suis* CC20 isolate reported from outside of the Netherlands [17]. With this finding, we corroborate a previously established ancestral link between the Dutch CC20 isolates and Chinese isolates [9].

**Figure 2:**
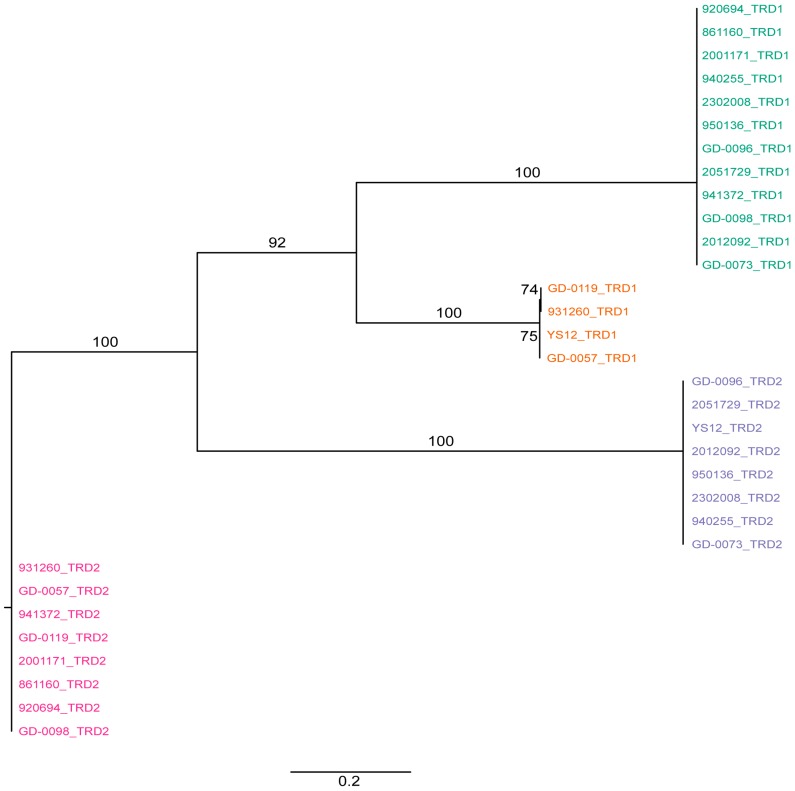
Phylogenetic tree of all TRDs from SsuCC20P as present in all CC20 isolates. Tips are labelled as isolates with the location of the TRD (first [TRD1] or second [TRD2] location in the S subunit) and are colored according to the position of the TRD in the genetic map in Figure 1B. TRD1s cluster together before clustering with TRD2s, suggesting that the TRDs have a fixed position in the gene, which limits the possible S subunits variants in SsuCC20P to the observed four variants. Bootstrap values >50% are indicated as percentages on the branches of the maximum likelihood tree, which was constructed with RAxML and are based on 1050 bootstraps.

We extracted the sequences of the TRDs from the *hsdS* genes to determine whether, within gene domain, movement occurred, similar to what was observed previously in *Helicobacter pylori* [19]. We found a conserved region in the nucleotide alignment between the TRD’s of 17 nucleotides in length (AGATAAATCATCATTTA) and two shorter conserved nucleotide sequences (ATTCAAAAGTTA and TCAGGCGA) at the 3′ end of the *hsdS* genes (Supplementary Figure S1). There was no evidence for repeat sequences in the S subunit, suggesting SsuCC20P is a Group 1 Type I RM system as defined by Furuta et al. [19]. This lack of repeat sequences further suggests that recombination occurs only within TRD1s and within TRD2s, but not across TRD1 and TRD2, within the four variants of SsuCC20P. This hypothesis is supported by phylogenetic analysis of all TRD1s and TRD2s (Figure 2), which shows clustering of TRDs.

To determine if this novel Type I R–M system is unique for the *S. suis* CC20 genomes, we searched for the presence of all Type I R–M systems in the Dutch 116 *S. suis* isolates under study. In homology groups, which were created previously [9], the presence of a Type I R subunit was searched for. An hmmsearch for the Type I restriction enzyme R protein N terminus protein family (HSDR_N, PF04313.10) was performed using a randomly selected protein representative for each of the homology groups. Five homology groups were found to possess Type I R subunit proteins. The R subunits found in four homology groups identified with three Type I R–M systems, as found in serotype 2 CC1 isolates (as described in the REBASE) and with the novel R–M system, identified in CC20 isolates (Table 1). The fifth group consisted of sequences from isolates TL13 and GD-0105, which are both singletons in the MLST scheme, and represented the R–M system Ssu13ORF242P as found in isolate TL13. The main four Type I R–M systems are distributed unevenly among the 116 isolates (Table 1) and four isolates (OV585, 6407, 9401240 and GD-0027) seem to completely lack Type I R–M systems.

SsuPORF1588P (we follow the gene numbering as was used for reference isolate P1/7 in REBASE) was found in 110 out of 116 isolates and could be considered a “core” Type I R–M system. Interestingly, SsuPORF1588P was found between a serine tRNA ligase (WP_012775316) and an *N*-acetyltransferase (WP_002938864) and included an integrase (WP_012028462) as well (Supplementary Figure S2). This suggests that SsuPORF1588P could be a prophage (remnant), but its GC content (41%) and the distribution of this system across the isolates suggest it was acquired a long time ago. SsuPORF1588P has an additional S subunit predicted in reference genomes (WP_012028461), but it should be noted that this S subunit does not include the pfam domains (Methylase_S and HsdS), which are commonly associated with S subunits. Whether the predicted integrase may facilitate TRD switching in the presence of one or two S subunits in SsuPORF1588P remains to be determined.

SsuPORF1273P was found in all CC1, CC16 and CC20 strains except for two CC16 isolates (9501632 and GD-0079), but not in CC13 and CC27/29 isolates. SsuPORF1273P has a low GC content of 36% and was found between a ribose-5-phosphate isomerase (WP_012027385) and a tRNA modification GTPase (WP_012775444) (Supplementary Figure S2). We found other insertions at this site in isolates 9501632, GD-0079 and in CC13 isolates (Supplementary Figure S3). SsuPORF1273P may therefore have a foreign origin as well. GD-0079 has a truncated R subunit of SsuPORF1273P, possibly rendering it a pseudogene, due to the insertion of an additional phage between the ribose-5-phosphate isomerase and the tRNA modification GTPase gene. The 9501632 only contains a hypothetical protein and a DEAD/DEAH box helicase at this site, whilst CC13 isolates have a phage inserted in the genome at the same location. SsuPORF1273P has access to two different S subunits, but lacks an integrase. CC1, CC16 and CC20 are the most common virulent isolates in the Netherlands [14] and it is interesting that these isolates contain an additional Type I R–M system.

Two additional Type I R–M systems—SsuPORF652P and SsuCC20P—were found among the *S. suis* isolates (Supplementary Figure S2). SsuPORF652P is likely to be a pseudogene, because the *hsdM* gene has a frameshift which was also annotated in P1/7. We found an M subunit without frameshifts in CC16 isolates, but the R subunit was truncated in these isolates. SsuPORF652P can be found in almost all CC1 isolates (except 98HAH33 and GZ1) and in selected isolates from CC16 (9501632, GD-0050 and GD-0063), CC20 (GD-0057, GD-0073 and GD-0098) and in a singleton; GD-0015. Coincidentally, 9501632, GD-0050 and GD-0063 are the only CC16 isolates that grouped together on a completely separate branch in the phylogenetic tree, away from other CC16 isolates (Supplementary Figure S4), while GD-0057, GD-0073 and GD-0098 group together as serotype 4 and ST17 isolates. SsuPORF652P Type I R–M system has access to a single S subunit.

The presence of any of the four variants of the SsuCC20P S subunits did not correlate with date of isolation or serotype. Three variants could be found among the serotype 2 ST20 isolates and we even found three variants in the three serotype 4 ST17 isolates. This observation indicates co-circulating S variants within the CC20 population. In addition, given the high genetic similarities between SsuCC20P and SpnD39III, the observed variation in S subunits suggests the possibility of rapid switching of S subunits within strains, as was shown for SpnD39III in *S. pneumoniae* [15]. However, the latter hypothesis as well as the potential effect of such subunit switching on gene regulation remains to be confirmed under experimental conditions. SsuCC20P, as described in this communication, is not present in serotype 2 isolates that belong to CC1, but our data suggest that the CC1 isolates do possess a different additional Type I R–M system: SsuPORF652P. However, the *hsdM* gene in SsuPORF652P appears to be a pseudo gene and only has access to one *hsdS* gene. Our data also suggest that the distribution of Type I R–M systems is associated with the clonal population structure of *S. suis*. Exceptions to this are the two CC16 isolates (GD-0050 and GD-0063) which form a distinct group amongst the CC16 population (Supplementary Figure S2) and have three Type I R–M systems instead of two. Additionally, the serotype 4 ST17 isolates, belonging to CC20, have four Type I R–M systems instead of three. We postulate that *S. suis* uses a ’core’ Type I R–M system (SsuPORF1588P) to protect itself from foreign DNA and that additional Type I R–M systems might allow for further phage defense and possibly for regulatory control. The presence of a ‘core’ Type I R–M system is further corroborated by the fact that SsuPORF1588P has a GC content of 41%, which is similar to the overall *S. suis* genome GC content of 41.3% [20], whilst the other Type I R–M systems have GC contents of 36%–39%. These differences are small and across short segments of DNA, but may suggest relatively recent acquisitions of these Type I R–M systems from foreign sources.

## 3. Materials and Methods

### 3.1. Bacterial Isolates

The bacterial isolates analyzed in this communication have previously been described [9,14]. Metadata for these isolates are attached in Supplementary Table S3.

### 3.2. Whole Genome Sequencing and Characterization of Isolates

Whole genome sequencing was performed as described previously [9]. Briefly, the isolates were sequenced using Illumina Miseq using 2 × 150 bp paired end reads and assembled using Spades 3.0.0 [21]. The isolates were genotyped into sequence type (ST) and subsequent clonal complexes (CC) using Multi Locus Sequence Typing (MLST) [22] and the eBurst algorithm [23]. Serotyping was performed by PCR or slide agglutination as described previously [14].

### 3.3. Restriction–Modification System Search

We used the HMMER package [24] in combination with the pfam database [25] to search for R–M systems in previously created orthology groups using OrthoMCL [26]. The hmmsearch command was used to search for the Type I restriction enzyme R protein N terminus (HSDR_N, pfam family PF04313.10) with a threshold for significant hits set at an E-value of 1 × 10^−3^. The HSDR_N is part of the R subunit of the R–M system and was chosen in favor to pfam families present in M and S subunits as M and S subunits can also be found as truncated and pseudogenes and can be missed by searching among orthology groups.

### 3.4. Phylogenetic Analysis of Target Recognition Domains

The S subunits were aligned at the amino acid level in seaview 4.3.1 [27] using Muscle v3.8.31 [28] and transformed back to a nucleotide alignment. The nucleotide alignment was split at the conserved region between the TRDs in TRD1 and TRD2 from which a phylogenetic tree was constructed using RAxML 8.6.1 [29] with a GTRGAMMA substitution matrix. Convergence at the bootstopping criterion was reached after 1050 bootstraps and the best tree was visualized with bootstrap support percentages using FigTree 1.4.2.

## 4. Conclusions

The distribution of R–M systems in *S. suis* remains largely unexplored since the efforts by Sekizaki et al. in the early 2000s and the epigenomic landscape of *S. suis* is still untouched. However, our mapping of Type I R-M systems illustrated recombination between S subunits in SsuCC20P and suggests the potential for other functions than protection against foreign DNA for these R-M systems. There are now over a 1000 publicly available *S. suis* genomes which are waiting to be explored. Additionally, new single molecule real-time sequencing (SMRT) techniques are available to study genome-wide methylation patterns. We propose a broad search for all R–M systems in *S. suis* without geographical restrictions and to investigate their potential implications for gene regulation and virulence.

## Figures and Tables

**Table 1:** Distribution of Type I R–M-systems across 98 Dutch *S. suis* genomes and 18 complete reference genomes.

Clonal Complex (Nr. of isolates)	SsuPORF1588P ^†^ Nr. of isolates	SsuPORF1273P ^†^ Nr. of isolates	SsuPORF652P ^†^ Nr. of isolates	SsuCC20P Nr. of isolates	Ssu13ORF242P ^†^ Nr. of isolates
CC1 (44)	44	44	42 ^a^	0	0
CC13 (7)	6 ^b^	0	0	0	0
CC16 (31)	31	29 ^c^	3 ^d^	0	0
CC20 (16)	16	16	3 ^e^	15 ^f^	0
CC27/CC29 (9)	9	0	0	0	0
Singleton (9)	4	2	1	0	2
Total (116)	110	91	49	15	2

^†^: R–M-systems are named according to their name in the Restriction Enzyme Database (REBASE) for isolate P1/7 except for Ssu13ORF242P which is named according to their name in isolate TL13; ^a^: Absent in 98HAH33 and GZ1; ^b^: Absent in OV585; ^c^: Absent in 9501632 and GD-0079; ^d^: Present in 9501632, GD-0050 and GD-0063; ^e^: Present in GD-0057, GD-0073 and GD-0098; ^f^: Absent in GD-0001.

## Supplementary Materials

The following are available online at www.mdpi.com/2076-0817/5/4/62/s1, Figure S1: Nucleotide sequence alignment of the full length S subunit of all isolates containing SsuCC20P, Figure S2: Genetic map of Type I R–M systems in *S. suis* except SsuCC20P, Figure S3: Genomic alignment of the insertions near the SsuPORF1273P Type I R–M system locus, Figure S4: Distribution of Type I R–M systems in *S. suis*, Table S1: Best blastp hits of genes present in the 18 kb prophage region as presented in Figure 1B. Table S2: Characteristics of isolates containing the prophage region and the Type I R–M system, Table S3: Characteristics of the *S. suis* isolates analyzed in this study.
