# Supplementary material for: Distribution of Type I Restriction–Modification Systems in Streptococcus suis: An Outlook
Source: Pathogens. 2016 Nov 18;5(4):62. doi: 10.3390/pathogens5040062 (PMC5198162; doi:10.3390/pathogens5040062)
Supplement: Supplementary file 1 [file pathogens-05-00062-s001.zip › figure_s1.pdf]

TRD1

```
861160     ------------------------TTGTCAGAAGTCGGTAGCTATGTCTCTGAAAAAATC
920694     ------------------------TTGTCAGAAGTCGGTAGCTATGTCTCTGAAAAAATC
941372     ------------------------TTGTCAGAAGTCGGTAGCTATGTCTCTGAAAAAATC
2001171    ------------------------TTGTCAGAAGTCGGTAGCTATGTCTCTGAAAAAATC
GD-0098    ------------------------TTGTCAGAAGTCGGTAGCTATGTCTCTGAAAAAATC
940255     ------------------------TTGTCAGAAGTCGGTAGCTATGTCTCTGAAAAAATC
950136     ------------------------TTGTCAGAAGTCGGTAGCTATGTCTCTGAAAAAATC
2012092    ------------------------TTGTCAGAAGTCGGTAGCTATGTCTCTGAAAAAATC
2302008    ------------------------TTGTCAGAAGTCGGTAGCTATGTCTCTGAAAAAATC
2051729    ------------------------TTGTCAGAAGTCGGTAGCTATGTCTCTGAAAAAATC
GD-0073    ------------------------TTGTCAGAAGTCGGTAGCTATGTCTCTGAAAAAATC
GD-0096    ------------------------TTGTCAGAAGTCGGTAGCTATGTCTCTGAAAAAATC
931260     TTGAACGATTGGAAATGGGTAAAGATTTCAGAAATCATAGATTTT---AATCCAAAAGAA
GD-0057    TTGAACGATTGGAAATGGGTAAAGCTTTCAGAAATCATAGATTTT---AATCCAAAAGAA
GD-0119    TTGAACGATTGGAAATGGGTAAAGATTTCAGAAATCATAGATTTT---AATCCAAAAGAA
YS12       TTGAACGATTGGAAATGGGTAAAGCTTTCAGAAATCATAGATTTT---AATCCAAAAGAA

861160     AATGTGAGTAAGTTAAGGCTGGAGAACTATATTTCTACCGAGAATATGATCTCTAATCGT
920694     AATGTGAGTAAGTTAAGGCTGGAGAACTATATTTCTACCGAGAATATGATCTCTAATCGT
941372     AATGTGAGTAAGTTAAGGCTGGAGAACTATATTTCTACCGAGAATATGATCTCTAATCGT
2001171    AATGTGAGTAAGTTAAGGCTGGAGAACTATATTTCTACCGAGAATATGATCTCTAATCGT
GD-0098    AATGTGAGTAAGTTAAGGCTGGAGAACTATATTTCTACCGAGAATATGATCTCTAATCGT
940255     AATGTGAGTAAGTTAAGGCTGGAGAACTATATTTCTACCGAGAATATGATCTCTAATCGT
950136     AATGTGAGTAAGTTAAGGCTGGAGAACTATATTTCTACCGAGAATATGATCTCTAATCGT
2012092    AATGTGAGTAAGTTAAGGCTGGAGAACTATATTTCTACCGAGAATATGATCTCTAATCGT
2302008    AATGTGAGTAAGTTAAGGCTGGAGAACTATATTTCTACCGAGAATATGATCTCTAATCGT
2051729    AATGTGAGTAAGTTAAGGCTGGAGAACTATATTTCTACCGAGAATATGATCTCTAATCGT
GD-0073    AATGTGAGTAAGTTAAGGCTGGAGAACTATATTTCTACCGAGAATATGATCTCTAATCGT
GD-0096    AATGTGAGTAAGTTAAGGCTGGAGAACTATATTTCTACCGAGAATATGATCTCTAATCGT
931260     CGGTTGCTTAAGGGAAGCATTTCTAAAAAAATAGCAATGGAAAAAATAGAACCATTCACT
GD-0057    CGGTTGCTTAAGGGAAGCATTTCTAAAAAAATAGCAATGGAAAAAATAGAACCATTCACT
GD-0119    CGGTTGCTTAAGGGAAGCATTTCTAAAAAAATAGCAATGGAAAAAATAGAACCATTCACT
YS12       CGGTTGCTTAAGGGAAGCATTTCTAAAAAAATAGCAATGGAAAAAATAGAACCATTCACT

861160     GGTGGAATTAGTTTGGCAACAAAACTTCCAAGTGTAAAAACTACAACTGCATTTCAAAAG
920694     GGTGGAATTAGTTTGGCAACAAAACTTCCAAGTGTAAAAACTACAACTGCATTTCAAAAG
941372     GGTGGAATTAGTTTGGCAACAAAACTTCCAAGTGTAAAAACTACAACTGCATTTCAAAAG
2001171    GGTGGAATTAGTTTGGCAACAAAACTTCCAAGTGTAAAAACTACAACTGCATTTCAAAAG
GD-0098    GGTGGAATTAGTTTGGCAACAAAACTTCCAAGTGTAAAAACTACAACTGCATTTCAAAAG
940255     GGTGGAATTAGTTTGGCAACAAAACTTCCAAGTGTAAAAACTACAACTGCATTTCAAAAG
950136     GGTGGAATTAGTTTGGCAACAAAACTTCCAAGTGTAAAAACTACAACTGCATTTCAAAAG
2012092    GGTGGAATTAGTTTGGCAACAAAACTTCCAAGTGTAAAAACTACAACTGCATTTCAAAAG
2302008    GGTGGAATTAGTTTGGCAACAAAACTTCCAAGTGTAAAAACTACAACTGCATTTCAAAAG
2051729    GGTGGAATTAGTTTGGCAACAAAACTTCCAAGTGTAAAAACTACAACTGCATTTCAAAAG
GD-0073    GGTGGAATTAGTTTGGCAACAAAACTTCCAAGTGTAAAAACTACAACTGCATTTCAAAAG
GD-0096    GGTGGAATTAGTTTGGCAACAAAACTTCCAAGTGTAAAAACTACAACTGCATTTCAAAAG
931260     CGTGATATTTCTGAGTTTGAGAGATTA---GAATTTAAAGGAGGTACAAAATTTAGAAAT
GD-0057    CGTGATATTTCTGAGTTTGAGAGATTA---GAATTTAAAGGAGGTACAAAATTTAGAAAT
GD-0119    CGTGATATTTCTGAGTTTGAGAGATTA---GAATTTAAAGGAGGTACAAAATTTAGAAAT
YS12       CGTGATATTTCTGAGTTTGAGAGATTA---GAATTTAAAGGAGGTACAAAATTTAGAAAT
```

```
861160   GGAGATATTCTAATTTCCAATATTCGCCCTTACTTTAAG---------------------
920694   GGAGATATTCTAATTTCCAATATTCGCCCTTACTTTAAG---------------------
941372   GGAGATATTCTAATTTCCAATATTCGCCCTTACTTTAAG---------------------
2001171  GGAGATATTCTAATTTCCAATATTCGCCCTTACTTTAAG---------------------
GD-0098  GGAGATATTCTAATTTCCAATATTCGCCCTTACTTTAAG---------------------
940255   GGAGATATTCTAATTTCCAATATTCGCCCTTACTTTAAG---------------------
950136   GGAGATATTCTAATTTCCAATATTCGCCCTTACTTTAAG---------------------
2012092  GGAGATATTCTAATTTCCAATATTCGCCCTTACTTTAAG---------------------
2302008  GGAGATATTCTAATTTCCAATATTCGCCCTTACTTTAAG---------------------
2051729  GGAGATATTCTAATTTCCAATATTCGCCCTTACTTTAAG---------------------
GD-0073  GGAGATATTCTAATTTCCAATATTCGCCCTTACTTTAAG---------------------
GD-0096  GGAGATATTCTAATTTCCAATATTCGCCCTTACTTTAAG---------------------
931260   GGCGATACTTTAATAGCCCGTATTACGCCCAGTCTTGAAAATGGGAAAACAGCTAAGGTA
GD-0057  GGCGATACTTTAATAGCCCGTATTACGCCCAGTCTTGAAAATGGGAAAACAGCTAAGGTA
GD-0119  GGCGATACTTTAATAGCCCGTATTACGCCCAGTCTTGAAAATGGGAAAACAGCTAAGGTA
YS12     GGCGATACTTTAATAGCCCGTATTACGCCCAGTCTTGAAAATGGGAAAACAGCTAAGGTA

861160   AAAATTTGGCTAGCAGACAAATCTGGAGGGTGTTCTAATGATGTTTTAGTAATCAGGTCG
920694   AAAATTTGGCTAGCAGACAAATCTGGAGGGTGTTCTAATGATGTTTTAGTAATCAGGTCG
941372   AAAATTTGGCTAGCAGACAAATCTGGAGGGTGTTCTAATGATGTTTTAGTAATCAGGTCG
2001171  AAAATTTGGCTAGCAGACAAATCTGGAGGGTGTTCTAATGATGTTTTAGTAATCAGGTCG
GD-0098  AAAATTTGGCTAGCAGACAAATCTGGAGGGTGTTCTAATGATGTTTTAGTAATCAGGTCG
940255   AAAATTTGGCTAGCAGACAAATCTGGAGGGTGTTCTAATGATGTTTTAGTAATCAGGTCG
950136   AAAATTTGGCTAGCAGACAAATCTGGAGGGTGTTCTAATGATGTTTTAGTAATCAGGTCG
2012092  AAAATTTGGCTAGCAGACAAATCTGGAGGGTGTTCTAATGATGTTTTAGTAATCAGGTCG
2302008  AAAATTTGGCTAGCAGACAAATCTGGAGGGTGTTCTAATGATGTTTTAGTAATCAGGTCG
2051729  AAAATTTGGCTAGCAGACAAATCTGGAGGGTGTTCTAATGATGTTTTAGTAATCAGGTCG
GD-0073  AAAATTTGGCTAGCAGACAAATCTGGAGGGTGTTCTAATGATGTTTTAGTAATCAGGTCG
GD-0096  AAAATTTGGCTAGCAGACAAATCTGGAGGGTGTTCTAATGATGTTTTAGTAATCAGGTCG
931260   AACTTACTGGATGAAGATGAAATAGGGTTTGGTTCAACAGAATTTATCGTAGCAAGAGCC
GD-0057  AACTTACTGGATGAAGATGAAATAGGGTTTGGTTCAACAGAATTTATCGTAGCAAGAGCC
GD-0119  AACTTACTGGATGAAGATGAAATAGGGTTTGGTTCAACAGAATTTATCGTAGCAAGAGCC
YS12     AACTTACTGGATGAAGATGAAATAGGGTTTGGTTCAACAGAATTTATCGTAGCAAGAGCC

861160   GACAGTAACTTTTCCAAC---CGTTTTCTATATTATGTGCTATCAAGTGATACATTTTTT
920694   GACAGTAACTTTTCCAAC---CGTTTTCTATATTATGTGCTATCAAGTGATACATTTTTT
941372   GACAGTAACTTTTCCAAC---CGTTTTCTATATTATGTGCTATCAAGTGATACATTTTTT
2001171  GACAGTAACTTTTCCAAC---CGTTTTCTATATTATGTGCTATCAAGTGATACATTTTTT
GD-0098  GACAGTAACTTTTCCAAC---CGTTTTCTATATTATGTGCTATCAAGTGATACATTTTTT
940255   GACAGTAACTTTTCCAAC---CGTTTTCTATATTATGTGCTATCAAGTGATACATTTTTT
950136   GACAGTAACTTTTCCAAC---CGTTTTCTATATTATGTGCTATCAAGTGATACATTTTTT
2012092  GACAGTAACTTTTCCAAC---CGTTTTCTATATTATGTGCTATCAAGTGATACATTTTTT
2302008  GACAGTAACTTTTCCAAC---CGTTTTCTATATTATGTGCTATCAAGTGATACATTTTTT
2051729  GACAGTAACTTTTCCAAC---CGTTTTCTATATTATGTGCTATCAAGTGATACATTTTTT
GD-0073  GACAGTAACTTTTCCAAC---CGTTTTCTATATTATGTGCTATCAAGTGATACATTTTTT
GD-0096  GACAGTAACTTTTCCAAC---CGTTTTCTATATTATGTGCTATCAAGTGATACATTTTTT
931260   AAAAAAGGTATTAGTGATGAAAATTTTGTTTATTATCTAATGCTTGACCCAAAGGTTAGA
GD-0057  AAAAAAGGTATTAGTGATGAAAATTTTGTTTATTATCTAATGCTTGACCCAAAGGTTAGA
GD-0119  AAAAAAGGTATTAGTGATGAAAATTTTGTTTATTATCTAATGCTTGACCCAAAGGTTAGA
YS12     AAAAAAGGTATTAGTGATGAAAATTTTGTTTATTATCTAATGCTTGACCCAAAGGTTAGA
```

```
861160    GATTATGCAGTATCAACTTCTAAGGGGACGAAG---ATGCCCCGTGGAGATAAGAGTTCA
920694    GATTATGCAGTATCAACTTCTAAGGGGACGAAG---ATGCCCCGTGGAGATAAGAGTTCA
941372    GATTATGCAGTATCAACTTCTAAGGGGACGAAG---ATGCCCCGTGGAGATAAGAGTTCA
2001171   GATTATGCAGTATCAACTTCTAAGGGGACGAAG---ATGCCCCGTGGAGATAAGAGTTCA
GD-0098   GATTATGCAGTATCAACTTCTAAGGGGACGAAG---ATGCCCCGTGGAGATAAGAGTTCA
940255    GATTATGCAGTATCAACTTCTAAGGGGACGAAG---ATGCCCCGTGGAGATAAGAGTTCA
950136    GATTATGCAGTATCAACTTCTAAGGGGACGAAG---ATGCCCCGTGGAGATAAGAGTTCA
2012092   GATTATGCAGTATCAACTTCTAAGGGGACGAAG---ATGCCCCGTGGAGATAAGAGTTCA
2302008   GATTATGCAGTATCAACTTCTAAGGGGACGAAG---ATGCCCCGTGGAGATAAGAGTTCA
2051729   GATTATGCAGTATCAACTTCTAAGGGGACGAAG---ATGCCCCGTGGAGATAAGAGTTCA
GD-0073   GATTATGCAGTATCAACTTCTAAGGGGACGAAG---ATGCCCCGTGGAGATAAGAGTTCA
GD-0096   GATTATGCAGTATCAACTTCTAAGGGGACGAAG---ATGCCCCGTGGAGATAAGAGTTCA
931260    GAAGTTGCTATCAAATCAATGGTAGGAACTTCTGGAAGACAAAGAGTTCAGCTAGATGTT
GD-0057   GAAGTTGCTATCAAATCAATGGTAGGAACTTCTGGAAGACAAAGAGTTCAGCTAGATGTT
GD-0119   GAAGTTGCTATCAAATCAATGGTAGGAACTTCTGGAAGACAAAGAGTTCAGCTAGATGTT
YS12      GAAGTTGCTATCAAATCAATGGTAGGAACTTCTGGAAGACAAAGAGTTCAGCTAGATGTT

861160    ATTATGAAATATACAGTTCCTTTATTTTCCTTAGAGGAGCAAGAGTTGATTTCAGATATA
920694    ATTATGAAATATACAGTTCCTTTATTTTCCTTAGAGGAGCAAGAGTTGATTTCAGATATA
941372    ATTATGAAATATACAGTTCCTTTATTTTCCTTAGAGGAGCAAGAGTTGATTTCAGATATA
2001171   ATTATGAAATATACAGTTCCTTTATTTTCCTTAGAGGAGCAAGAGTTGATTTCAGATATA
GD-0098   ATTATGAAATATACAGTTCCTTTATTTTCCTTAGAGGAGCAAGAGTTGATTTCAGATATA
940255    ATTATGAAATATACAGTTCCTTTATTTTCCTTAGAGGAGCAAGAGTTGATTTCAGATATA
950136    ATTATGAAATATACAGTTCCTTTATTTTCCTTAGAGGAGCAAGAGTTGATTTCAGATATA
2012092   ATTATGAAATATACAGTTCCTTTATTTTCCTTAGAGGAGCAAGAGTTGATTTCAGATATA
2302008   ATTATGAAATATACAGTTCCTTTATTTTCCTTAGAGGAGCAAGAGTTGATTTCAGATATA
2051729   ATTATGAAATATACAGTTCCTTTATTTTCCTTAGAGGAGCAAGAGTTGATTTCAGATATA
GD-0073   ATTATGAAATATACAGTTCCTTTATTTTCCTTAGAGGAGCAAGAGTTGATTTCAGATATA
GD-0096   ATTATGAAATATACAGTTCCTTTATTTTCCTTAGAGGAGCAAGAGTTGATTTCAGATATA
931260    GTTCAGAATTACGAAATTCTATGTCCTCCATTAGAGGAGCAAATTCAAATTGGCAGAATT
GD-0057   GTTCAGAATTACGAAATTCTATGTCCTCCATTAGAGGAGCAAATTCAAATTGGCAGAATT
GD-0119   GTTCAGAATTACGAAATTCTATGTCCTCCATTAGAGGAGCAAATTCAAATTGGCAGAATT
YS12      GTTCAGAATTACGAAATTCTATGTCCTCCATTAGAGGAGCAAATTCAAATTGGCAGAATT

                                                   Conserved region TRD2

861160    TTGAAGTCCTATGACGAGAAAATTAAACTTAATAAACAGATAAATCATCATTTACTTGAG
920694    TTGAAGTCCTATGACGAGAAAATTAAACTTAATAAACAGATAAATCATCATTTACTTGAG
941372    TTGAAGTCCTATGACGAGAAAATTAAACTTAATAAACAGATAAATCATCATTTACTTGAG
2001171   TTGAAGTCCTATGACGAGAAAATTAAACTTAATAAACAGATAAATCATCATTTACTTGAG
GD-0098   TTGAAGTCCTATGACGAGAAAATTAAACTTAATAAACAGATAAATCATCATTTACTTGAG
940255    TTGAAGTCCTATGACGAGAAAATTAAACTTAATAAACAGATAAATCATCATTTAGCTGAG
950136    TTGAAGTCCTATGACGAGAAAATTAAACTTAATAAACAGATAAATCATCATTTAGCTGAG
2012092   TTGAAGTCCTATGACGAGAAAATTAAACTTAATAAACAGATAAATCATCATTTAGCTGAG
2302008   TTGAAGTCCTATGACGAGAAAATTAAACTTAATAAACAGATAAATCATCATTTAGCTGAG
2051729   TTGAAGTCCTATGACGAGAAAATTAAACTTAATAAACAGATAAATCATCATTTAGCTGAG
GD-0073   TTGAAGTCCTATGACGAGAAAATTAAACTTAATAAACAGATAAATCATCATTTAGCTGAG
GD-0096   TTGAAGTCCTATGACGAGAAAATTAAACTTAATAAACAGATAAATCATCATTTAGCTGAG
931260    TTGAGTGTAATTGATGATAAAATTGAAAATAATAAAAGATAAATCATCATTTACTTGAG
GD-0057   TTGAGTGTAATTGATGATAAAATTGAAAATAATAAAAGATAAATCATCATTTACTTGAG
GD-0119   TTGAGTGTAATTGATGATAAAATTGAAAATAATAAAAGATAAATCATCATTTACTTGAG
YS12      TTGAGTGTAATTGATGATAAAATTGAAAATAATAAAAGATAAATCATCATTTAGCTGAG
```

```
861160     CAAGCTAGATTGCTTTATAAAAATTTAATATCGTCTAATGACACTAAATATCAAAATCTT
920694     CAAGCTAGATTGCTTTATAAAAATTTAATATCGTCTAATGACACTAAATATCAAAATCTT
941372     CAAGCTAGATTGCTTTATAAAAATTTAATATCGTCTAATGACACTAAATATCAAAATCTT
2001171    CAAGCTAGATTGCTTTATAAAAATTTAATATCGTCTAATGACACTAAATATCAAAATCTT
GD-0098    CAAGCTAGATTGCTTTATAAAAATTTAATATCGTCTAATGACACTAAATATCAAAATCTT
940255     CTAATCGATGCTCAATTTGCTCACTTGCTTGAAGATAATGAATTATATAAGTCGACCTTT
950136     CTAATCGATGCTCAATTTGCTCACTTGCTTGAAGATAATGAATTATATAAGTCGACCTTT
2012092    CTAATCGATGCTCAATTTGCTCACTTGCTTGAAGATAATGAATTATATAAGTCGACCTTT
2302008    CTAATCGATGCTCAATTTGCTCACTTGCTTGAAGATAATGAATTATATAAGTCGACCTTT
2051729    CTAATCGATGCTCAATTTGCTCACTTGCTTGAAGATAATGAATTATATAAGTCGACCTTT
GD-0073    CTAATCGATGCTCAATTTGCTCACTTGCTTGAAGATAATGAATTATATAAGTCGACCTTT
GD-0096    CTAATCGATGCTCAATTTGCTCACTTGCTTGAAGATAATGAATTATATAAGTCGACCTTT
931260     CAAGCTAGATTGCTTTATAAAAATTTAATATCGTCTAATGACACTAAATATCAAAATCTT
GD-0057    CAAGCTAGATTGCTTTATAAAAATTTAATATCGTCTAATGACACTAAATATCAAAATCTT
GD-0119    CAAGCTAGATTGCTTTATAAAAATTTAATATCGTCTAATGACACTAAATATCAAAATCTT
YS12       CTAATCGATGCTCAATTTGCTCACTTGCTTGAAGATAATGAATTATATAAGTCGACCTTT

861160     TCAGATATTGCTAGAATTACGATGGGACAATCTCCTAAG---------GGTGAGACTTAT
920694     TCAGATATTGCTAGAATTACGATGGGACAATCTCCTAAG---------GGTGAGACTTAT
941372     TCAGATATTGCTAGAATTACGATGGGACAATCTCCTAAG---------GGTGAGACTTAT
2001171    TCAGATATTGCTAGAATTACGATGGGACAATCTCCTAAG---------GGTGAGACTTAT
GD-0098    TCAGATATTGCTAGAATTACGATGGGACAATCTCCTAAG---------GGTGAGACTTAT
940255     TCAGAAATTGGTGAAATAGTGGGCGGTGGTACGCCATCTAAAAAGGTAGATGATTACTGG
950136     TCAGAAATTGGTGAAATAGTGGGCGGTGGTACGCCATCTAAAAAGGTAGATGATTACTGG
2012092    TCAGAAATTGGTGAAATAGTGGGCGGTGGTACGCCATCTAAAAAGGTAGATGATTACTGG
2302008    TCAGAAATTGGTGAAATAGTGGGCGGTGGTACGCCATCTAAAAAGGTAGATGATTACTGG
2051729    TCAGAAATTGGTGAAATAGTGGGCGGTGGTACGCCATCTAAAAAGGTAGATGATTACTGG
GD-0073    TCAGAAATTGGTGAAATAGTGGGCGGTGGTACGCCATCTAAAAAGGTAGATGATTACTGG
GD-0096    TCAGAAATTGGTGAAATAGTGGGCGGTGGTACGCCATCTAAAAAGGTAGATGATTACTGG
931260     TCAGATATTGCTAGAATTACGATGGGACAATCTCCTAAG---------GGTGAGACTTAT
GD-0057    TCAGATATTGCTAGAATTACGATGGGACAATCTCCTAAG---------GGTGAGACTTAT
GD-0119    TCAGATATTGCTAGAATTACGATGGGACAATCTCCTAAG---------GGTGAGACTTAT
YS12       TCAGAAATTGGTGAAATAGTGGGCGGTGGTACGCCATCTAAAAAGGTAGATGATTACTGG

861160     AATGATGAC---------------------------AAGATTGGTTTGCCGTTATTAAAT
920694     AATGATGAC---------------------------AAGATTGGTTTGCCGTTATTAAAT
941372     AATGATGAC---------------------------AAGATTGGTTTGCCGTTATTAAAT
2001171    AATGATGAC---------------------------AAGATTGGTTTGCCGTTATTAAAT
GD-0098    AATGATGAC---------------------------AAGATTGGTTTGCCGTTATTAAAT
940255     AATGGTGATATCCCATGGCTTTCTCCAAAAGATTTATCATTAAATCCTGCTATGTTTACA
950136     AATGGTGATATCCCATGGCTTTCTCCAAAAGATTTATCATTAAATCCTGCTATGTTTACA
2012092    AATGGTGATATCCCATGGCTTTCTCCAAAAGATTTATCATTAAATCCTGCTATGTTTACA
2302008    AATGGTGATATCCCATGGCTTTCTCCAAAAGATTTATCATTAAATCCTGCTATGTTTACA
2051729    AATGGTGATATCCCATGGCTTTCTCCAAAAGATTTATCATTAAATCCTGCTATGTTTACA
GD-0073    AATGGTGATATCCCATGGCTTTCTCCAAAAGATTTATCATTAAATCCTGCTATGTTTACA
GD-0096    AATGGTGATATCCCATGGCTTTCTCCAAAAGATTTATCATTAAATCCTGCTATGTTTACA
931260     AATGATGAC---------------------------AAGATTGGTTTGCCGTTATTAAAT
GD-0057    AATGATGAC---------------------------AAGATTGGTTTGCCGTTATTAAAT
GD-0119    AATGATGAC---------------------------AAGATTGGTTTGCCGTTATTAAAT
YS12       AATGGTGATATCCCATGGCTTTCTCCAAAAGATTTATCATTAAATCCTGCTATGTTTACA
```

```
861160    GGAGCTACAGACTTCAGAAACAGTATTTCACCCTCAAAATGGACATCTGATCCTAGAAAA
920694    GGAGCTACAGACTTCAGAAACAGTATTTCACCCTCAAAATGGACATCTGATCCTAGAAAA
941372    GGAGCTACAGACTTCAGAAACAGTATTTCACCCTCAAAATGGACATCTGATCCTAGAAAA
2001171   GGAGCTACAGACTTCAGAAACAGTATTTCACCCTCAAAATGGACATCTGATCCTAGAAAA
GD-0098   GGAGCTACAGACTTCAGAAACAGTATTTCACCCTCAAAATGGACATCTGATCCTAGAAAA
940255    GGTAGAGGT------CAAAATTCAATCACTGAGCTTGGCTATAAGAAGAGCAGTGCTAAG
950136    GGTAGAGGT------CAAAATTCAATCACTGAGCTTGGCTATAAGAAGAGCAGTGCTAAG
2012092   GGTAGAGGT------CAAAATTCAATCACTGAGCTTGGCTATAAGAAGAGCAGTGCTAAG
2302008   GGTAGAGGT------CAAAATTCAATCACTGAGCTTGGCTATAAGAAGAGCAGTGCTAAG
2051729   GGTAGAGGT------CAAAATTCAATCACTGAGCTTGGCTATAAGAAGAGCAGTGCTAAG
GD-0073   GGTAGAGGT------CAAAATTCAATCACTGAGCTTGGCTATAAGAAGAGCAGTGCTAAG
GD-0096   GGTAGAGGT------CAAAATTCAATCACTGAGCTTGGCTATAAGAAGAGCAGTGCTAAG
931260    GGAGCTACAGACTTCAGAAACAGTATTTCACCCTCAAAATGGACATCTGATCCTAGAAAA
GD-0057   GGAGCTACAGACTTCAGAAACAGTATTTCACCCTCAAAATGGACATCTGATCCTAGAAAA
GD-0119   GGAGCTACAGACTTCAGAAACAGTATTTCACCCTCAAAATGGACATCTGATCCTAGAAAA
YS12      GGTAGAGGT------CAAAATTCAATCACTGAGCTTGGCTATAAGAAGAGCAGTGCTAAG
```

```
861160    ATAGCTAGACCTGGAGAATATGTGTTTGGTGTGAGAGCAACTATTGGTTTAACAACAAAA
920694    ATAGCTAGACCTGGAGAATATGTGTTTGGTGTGAGAGCAACTATTGGTTTAACAACAAAA
941372    ATAGCTAGACCTGGAGAATATGTGTTTGGTGTGAGAGCAACTATTGGTTTAACAACAAAA
2001171   ATAGCTAGACCTGGAGAATATGTGTTTGGTGTGAGAGCAACTATTGGTTTAACAACAAAA
GD-0098   ATAGCTAGACCTGGAGAATATGTGTTTGGTGTGAGAGCAACTATTGGTTTAACAACAAAA
940255    TTAATGCCTCGGAATTCAATACTTTTCAGTTCCCGCGCTCCGATTGGTTATATCACCATA
950136    TTAATGCCTCGGAATTCAATACTTTTCAGTTCCCGCGCTCCGATTGGTTATATCACCATA
2012092   TTAATGCCTCGGAATTCAATACTTTTCAGTTCCCGCGCTCCGATTGGTTATATCACCATA
2302008   TTAATGCCTCGGAATTCAATACTTTTCAGTTCCCGCGCTCCGATTGGTTATATCACCATA
2051729   TTAATGCCTCGGAATTCAATACTTTTCAGTTCCCGCGCTCCGATTGGTTATATCACCATA
GD-0073   TTAATGCCTCGGAATTCAATACTTTTCAGTTCCCGCGCTCCGATTGGTTATATCACCATA
GD-0096   TTAATGCCTCGGAATTCAATACTTTTCAGTTCCCGCGCTCCGATTGGTTATATCACCATA
931260    ATAGCTAGACCTGGAGAATATGTGTTTGGTGTGAGAGCAACTATTGGTTTAACAACAAAA
GD-0057   ATAGCTAGACCTGGAGAATATGTGTTTGGTGTGAGAGCAACTATTGGTTTAACAACAAAA
GD-0119   ATAGCTAGACCTGGAGAATATGTGTTTGGTGTGAGAGCAACTATTGGTTTAACAACAAAA
YS12      TTAATGCCTCGGAATTCAATACTTTTCAGTTCCCGCGCTCCGATTGGTTATATCACCATA
```

```
861160    ATATTTAAAGAATATGCTATTGGTCGAGGAACAGGTAGTGCAAAACCAATTTCTAATATC
920694    ATATTTAAAGAATATGCTATTGGTCGAGGAACAGGTAGTGCAAAACCAATTTCTAATATC
941372    ATATTTAAAGAATATGCTATTGGTCGAGGAACAGGTAGTGCAAAACCAATTTCTAATATC
2001171   ATATTTAAAGAATATGCTATTGGTCGAGGAACAGGTAGTGCAAAACCAATTTCTAATATC
GD-0098   ATATTTAAAGAATATGCTATTGGTCGAGGAACAGGTAGTGCAAAACCAATTTCTAATATC
940255    GCAGAAAATGATATTTCAACTAACCAAGGTTTTAAGTCAATCATTCCCAAACCTGAATAC
950136    GCAGAAAATGATATTTCAACTAACCAAGGTTTTAAGTCAATCATTCCCAAACCTGAATAC
2012092   GCAGAAAATGATATTTCAACTAACCAAGGTTTTAAGTCAATCATTCCCAAACCTGAATAC
2302008   GCAGAAAATGATATTTCAACTAACCAAGGTTTTAAGTCAATCATTCCCAAACCTGAATAC
2051729   GCAGAAAATGATATTTCAACTAACCAAGGTTTTAAGTCAATCATTCCCAAACCTGAATAC
GD-0073   GCAGAAAATGATATTTCAACTAACCAAGGTTTTAAGTCAATCATTCCCAAACCTGAATAC
GD-0096   GCAGAAAATGATATTTCAACTAACCAAGGTTTTAAGTCAATCATTCCCAAACCTGAATAC
931260    ATATTTAAAGAATATGCTATTGGTCGAGGAACAGGTAGTGCAAAACCAATTTCTAATATC
GD-0057   ATATTTAAAGAATATGCTATTGGTCGAGGAACAGGTAGTGCAAAACCAATTTCTAATATC
GD-0119   ATATTTAAAGAATATGCTATTGGTCGAGGAACAGGTAGTGCAAAACCAATTTCTAATATC
YS12      GCAGAAAATGATATTTCAACTAACCAAGGTTTTAAGTCAATCATTCCCAAACCTGAATAC
```

```
861160     TTTGATGAATATTTATTTTTCGCTTTGGAAGACCTTTTTGATTACTATGCTAATCTTGGT
920694     TTTGATGAATATTTATTTTTCGCTTTGGAAGACCTTTTTGATTACTATGCTAATCTTGGT
941372     TTTGATGAATATTTATTTTTCGCTTTGGAAGACCTTTTTGATTACTATGCTAATCTTGGT
2001171    TTTGATGAATATTTATTTTTCGCTTTGGAAGACCTTTTTGATTACTATGCTAATCTTGGT
GD-0098    TTTGATGAATATTTATTTTTCGCTTTGGAAGACCTTTTTGATTACTATGCTAATCTTGGT
940255     CCATATACTTTCGTGTATGAACTCTTGAAACAGGAAACTCCTTCCCTCGAGAGTAGTGCT
950136     CCATATACTTTCGTGTATGAACTCTTGAAACAGGAAACTCCTTCCCTCGAGAGTAGTGCT
2012092    CCATATACTTTCGTGTATGAACTCTTGAAACAGGAAACTCCTTCCCTCGAGAGTAGTGCT
2302008    CCATATACTTTCGTGTATGAACTCTTGAAACAGGAAACTCCTTCCCTCGAGAGTAGTGCT
2051729    CCATATACTTTCGTGTATGAACTCTTGAAACAGGAAACTCCTTCCCTCGAGAGTAGTGCT
GD-0073    CCATATACTTTCGTGTATGAACTCTTGAAACAGGAAACTCCTTCCCTCGAGAGTAGTGCT
GD-0096    CCATATACTTTCGTGTATGAACTCTTGAAACAGGAAACTCCTTCCCTCGAGAGTAGTGCT
931260     TTTGATGAATATTTATTTTTCGCTTTGGAAGACCTTTTTGATTACTATGCTAATCTTGGT
GD-0057    TTTGATGAATATTTATTTTTCGCTTTGGAAGACCTTTTTGATTACTATGCTAATCTTGGT
GD-0119    TTTGATGAATATTTATTTTTCGCTTTGGAAGACCTTTTTGATTACTATGCTAATCTTGGT
YS12       CCATATACTTTCGTGTATGAACTCTTGAAACAGGAAACTCCTTCCCTCGAGAGTAGTGCT

861160     TCGGGAACAGTTTATATAAATATTTCAAAATCTGATTTCGATAGCTTCAAAGTTATTCTT
920694     TCGGGAACAGTTTATATAAATATTTCAAAATCTGATTTCGATAGCTTCAAAGTTATTCTT
941372     TCGGGAACAGTTTATATAAATATTTCAAAATCTGATTTCGATAGCTTCAAAGTTATTCTT
2001171    TCGGGAACAGTTTATATAAATATTTCAAAATCTGATTTCGATAGCTTCAAAGTTATTCTT
GD-0098    TCGGGAACAGTTTATATAAATATTTCAAAATCTGATTTCGATAGCTTCAAAGTTATTCTT
940255     TCAGGTTCTACATTTAAAGAGGTATCTGGAACTCATTTGAAGAACCATGAGATTCGAATT
950136     TCAGGTTCTACATTTAAAGAGGTATCTGGAACTCATTTGAAGAACCATGAGATTCGAATT
2012092    TCAGGTTCTACATTTAAAGAGGTATCTGGAACTCATTTGAAGAACCATGAGATTCGAATT
2302008    TCAGGTTCTACATTTAAAGAGGTATCTGGAACTCATTTGAAGAACCATGAGATTCGAATT
2051729    TCAGGTTCTACATTTAAAGAGGTATCTGGAACTCATTTGAAGAACCATGAGATTCGAATT
GD-0073    TCAGGTTCTACATTTAAAGAGGTATCTGGAACTCATTTGAAGAACCATGAGATTCGAATT
GD-0096    TCAGGTTCTACATTTAAAGAGGTATCTGGAACTCATTTGAAGAACCATGAGATTCGAATT
931260     TCGGGAACAGTTTATATAAATATTTCAAAATCTGATTTCGATAGCTTCAAAGTTATTCTT
GD-0057    TCGGGAACAGTTTATATAAATATTTCAAAATCTGATTTCGATAGCTTCAAAGTTATTCTT
GD-0119    TCGGGAACAGTTTATATAAATATTTCAAAATCTGATTTCGATAGCTTCAAAGTTATTCTT
YS12       TCAGGTTCTACATTTAAAGAGGTATCTGGAACTCATTTGAAGAACCATGAGATTCGAATT

861160     CCAATAAAAGATCAATTTTTAGTTGACTTCCATAAAACAGTGCAACCATTATTTAATTTA
920694     CCAATAAAAGATCAATTTTTAGTTGACTTCCATAAAACAGTGCAACCATTATTTAATTTA
941372     CCAATAAAAGATCAATTTTTAGTTGACTTCCATAAAACAGTGCAACCATTATTTAATTTA
2001171    CCAATAAAAGATCAATTTTTAGTTGACTTCCATAAAACAGTGCAACCATTATTTAATTTA
GD-0098    CCAATAAAAGATCAATTTTTAGTTGACTTCCATAAAACAGTGCAACCATTATTTAATTTA
940255     CCG---CCCCATTCAGCAATAATTAAGTTTCATGAGTCAGTTGAGCCACTATTCAAGACA
950136     CCG---CCCCATTCAGCAATAATTAAGTTTCATGAGTCAGTTGAGCCACTATTCAAGACA
2012092    CCG---CCCCATTCAGCAATAATTAAGTTTCATGAGTCAGTTGAGCCACTATTCAAGACA
2302008    CCG---CCCCATTCAGCAATAATTAAGTTTCATGAGTCAGTTGAGCCACTATTCAAGACA
2051729    CCG---CCCCATTCAGCAATAATTAAGTTTCATGAGTCAGTTGAGCCACTATTCAAGACA
GD-0073    CCG---CCCCATTCAGCAATAATTAAGTTTCATGAGTCAGTTGAGCCACTATTCAAGACA
GD-0096    CCG---CCCCATTCAGCAATAATTAAGTTTCATGAGTCAGTTGAGCCACTATTCAAGACA
931260     CCAATAAAAGATCAATTTTTAGTTGACTTCCATAAAACAGTGCAACCATTATTTAATTTA
GD-0057    CCAATAAAAGATCAATTTTTAGTTGACTTCCATAAAACAGTGCAACCATTATTTAATTTA
GD-0119    CCAATAAAAGATCAATTTTTAGTTGACTTCCATAAAACAGTGCAACCATTATTTAATTTA
YS12       CCG---CCCCATTCAGCAATAATTAAGTTTCATGAGTCAGTTGAGCCACTATTCAAGACA
```

```
                                    Cons.Reg.

861160     ATATTTAATAATAATGCTGAAATTCAAAAGTTATCCGAGTTAAGGGATTGCCTCTTGCCA
920694     ATATTTAATAATAATGCTGAAATTCAAAAGTTATCCGAGTTAAGGGATTGCCTCTTGCCA
941372     ATATTTAATAATAATGCTGAAATTCAAAAGTTATCCGAGTTAAGGGATTGCCTCTTGCCA
2001171    ATATTTAATAATAATGCTGAAATTCAAAAGTTATCCGAGTTAAGGGATTGCCTCTTGCCA
GD-0098    ATATTTAATAATAATGCTGAAATTCAAAAGTTATCCGAGTTAAGGGATTGCCTCTTGCCA
940255     ATTAATTTGAATGAGAAAGAGATTCAAAAGTTAATCGAAGTGCGCGATCTTTTACTACCA
950136     ATTAATTTGAATGAGAAAGAGATTCAAAAGTTAATCGAAGTGCGCGATCTTTTACTACCA
2012092    ATTAATTTGAATGAGAAAGAGATTCAAAAGTTAATCGAAGTGCGCGATCTTTTACTACCA
2302008    ATTAATTTGAATGAGAAAGAGATTCAAAAGTTAATCGAAGTGCGCGATCTTTTACTACCA
2051729    ATTAATTTGAATGAGAAAGAGATTCAAAAGTTAATCGAAGTGCGCGATCTTTTACTACCA
GD-0073    ATTAATTTGAATGAGAAAGAGATTCAAAAGTTAATCGAAGTGCGCGATCTTTTACTACCA
GD-0096    ATTAATTTGAATGAGAAAGAGATTCAAAAGTTAATCGAAGTGCGCGATCTTTTACTACCA
931260     ATATTTAATAATAATGCTGAAATTCAAAAGTTATCCGAGTTAAGGGATTGCCTCTTGCCA
GD-0057    ATATTTAATAATAATGCTGAAATTCAAAAGTTATCCGAGTTAAGGGATTGCCTCTTGCCA
GD-0119    ATATTTAATAATAATGCTGAAATTCAAAAGTTATCCGAGTTAAGGGATTGCCTCTTGCCA
YS12       ATTAATTTGAATGAGAAAGAGATTCAAAAGTTAATCGAAGTGCGCGATCTTTTACTACCA

                      Cons.Reg.

861160     AAACTCCTATCAGGCGAAATTTCCATAAATCAAGCCACTAAATGA
920694     AAACTCCTATCAGGCGAAATTTCCATAAATCAAGCCACTAAATGA
941372     AAACTCCTATCAGGCGAAATTTCCATAAATCAAGCCACTAAATGA
2001171    AAACTCCTATCAGGCGAAATTTCCATAAATCAAGCCACTAAATGA
GD-0098    AAACTCCTATCAGGCGAAATTTCCATAAATCAAGCCACTAAATGA
940255     ACGCTTATGTCAGGCGAAATATCAGTCTCTGATTAA---------
950136     ACGCTTATGTCAGGCGAAATATCAGTCTCTGATTAA---------
2012092    ACGCTTATGTCAGGCGAAATATCAGTCTCTGATTAA---------
2302008    ACGCTTATGTCAGGCGAAATATCAGTCTCTGATTAA---------
2051729    ACGCTTATGTCAGGCGAAATATCAGTCTCTGATTAA---------
GD-0073    ACGCTTATGTCAGGCGAAATATCAGTCTCTGATTAA---------
GD-0096    ACGCTTATGTCAGGCGAAATATCAGTCTCTGATTAA---------
931260     AAACTCCTATCAGGCGAAATTTCCATAAATCAAGCCACTAAATGA
GD-0057    AAACTCCTATCAGGCGAAATTTCCATAAATCAAGCCACTAAATGA
GD-0119    AAACTCCTATCAGGCGATATTTCCATAAATCAAGCCACTAAATGA
YS12       ACGCTTATGTCAGGCGAAATATCAGTCTCTGATTAA---------
```
