# Supplementary material for: Distribution of Type I Restriction–Modification Systems in Streptococcus suis: An Outlook
Source: Pathogens. 2016 Nov 18;5(4):62. doi: 10.3390/pathogens5040062 (PMC5198162; doi:10.3390/pathogens5040062)
Supplement: Supplementary file 1 [file pathogens-05-00062-s001.zip › supplementary-legends-revised-2-final.pdf]

Supplementary Materials: Distribution of Type I Restriction-Modification Systems in *Streptococcus suis*: An Outlook

Niels Willemse and Constance Schultsz

Figure S1. Nucleotides sequences alignment of the *hsdS* genes from SsuCC20P present in all CC20 isolates. Target Recognition Domains (TRDs) are indicated above the nucleotide alignment and conserved nucleotides sequences across all isolates are indicated by black lines. The absence of repeat sequences and the position of the conserved domain indicate SsuCC20P is a group 1 Type I R-M system [1].

Figure S2. Genetic map of Type I R-M systems in *S. suis* except SsuCC20P. SsuPORF1588P, SsuPORF1273P and SsuPORF652P and their flanking region were extracted from isolate P1/7 (NC_012925) and Ssu13ORF242P was extracted from isolate TL13 (NC_021213) and subsequently visualized using Artemis [2]. The visualized open reading frames are P1/7: SsuPORF1588P; SSU1587 - SSU1596, SsuPORF1273P; SSU1270 - SSU1275, SsuPORF652P; SSU0650 - SSU0654, and TL13: Ssu13ORF242P; TL13_0241 - TL13_0248. Open reading frames were annotated with their best blastp hits: *hypothetical*: hypothetical protein, *int*: site-specific integrase, *N-acetyl*: N-acetyltransferase, *peptidoglycan*: peptidoglycan hydrolase, *pflC*: formate acetyltransferase, *pyr*: pyridine nucleotide-disulfide oxidoreductase, *rpiA*: ribose-5-phosphate isomerase, *serS*: serine--tRNA ligase, *trmE*: tRNA modification GTPase, #: contains a frameshift after codon 326, *: probable gene remnant. CDS is truncated at the N-terminus compared to similar proteins. ^: Predicted as a *hsdS* gene, but lacks the associated pfam domains HsdS and Methylase_S.

Figure S3. Genomic alignment of the insertions near the SsuPORF1273P Type I R-M system locus as illustrated in Supplementary Figure 3. Genomic loci were extracted from isolates GD-0079, P1/7, 9501632 and ST1 and aligned using the Artemis Comparison Tool [3] to illustrate the insertions inside SsuPORF1273P. Recombination between hsdS subunits can be seen in GD-0079 as well as a truncated *hsdR* gene and an additional DNA fragment, likely of phage origin. 9501632 shows the presence of a hypothetical protein and a DEAD/DEAH box helicase instead of SsuPORF1273P. Finally, ST1 is representative for CC13 isolates and demonstrates the complete absence of SsuPORF1273P, but an insertion of foreign DNA with a phage origin, which likely has the same origin as the phage DNA in GD-0079. Cyan: *rpiA* and *trmE* genes respectively as shown in Supplementary Figure 3, Blue: *hsd* genes making up the SsuPORF1273P Type I R-M system.

Figure S4. Distribution of Type I R-M systems in *S. suis*. A phylogenetic tree was created as previously [4]. The tree branches are overlaid with previously determined BAPS population groups and the clonal complex, serotype and host data for each isolate is indicated with a colored strip. The presence and absence for the five R-M systems is indicated by respectively solid and empty circles. The circles are colored by clonal complex. This tree was generated using the interative Tree of Life tool (iTOL): .itol.embl.de

Table S1. Annotation of the entire prophage region on which the SsuCC20P R-M resides. Genes are annotated from 5′ to 3′ according to order of the prophage in Figure 1B. Best hits from blastp with the identity and coverage percentage and the most likely function are presented.

Table S2. Characteristics of isolates containing the prophage region and the Type I R-M system.

Table S3. Characteristics of the *S. suis* isolates analyzed in this study. This table is similar to a previously supplementary table accompanying a paper by Willemse et al. except the presence and absence of the identified Type I R-M systems are also indicated here.

References

1. Furuta, Y.; Kawai, M.; Uchiyama, I.; Kobayashi, I. Domain movement within a gene: A novel evolutionary mechanism for protein diversification. *PLoS One* **2011**, *6*, e18819.
2. Rutherford, K.; Parkhill, J.; Crook, J.; Horsnell, T.; Rice, P.; Rajandream, M.A.; Barrell, B. Artemis: Sequence visualization and annotation. *Bioinformatics* **2000**, *16*, 944-945.
3. Carver, T.J.; Rutherford, K.M.; Berriman, M.; Rajandream, M.A.; Barrell, B.G.; Parkhill, J. ACT: The Artemis comparison tool. *Bioinformatics* **2005**, *21*, 3422-3423.
4. Willemse, N.; Howell, K.J.; Weinert, L.A.; Heuvelink, A.; Pannekoek, Y.; Wagenaar, J.A.; Smith, H.E.; van der Ende, A.; Schultsz, C. An emerging zoonotic clone in the Netherlands provides clues to virulence and zoonotic potential of *Streptococcus suis*. *Scientific reports* **2016**, *6*, 28984.
