# Supplementary figures and images for: Distribution of Type I Restriction–Modification Systems in Streptococcus suis: An Outlook
Source: Pathogens. 2016 Nov 18;5(4):62. doi: 10.3390/pathogens5040062 (PMC5198162; doi:10.3390/pathogens5040062)

P1/7

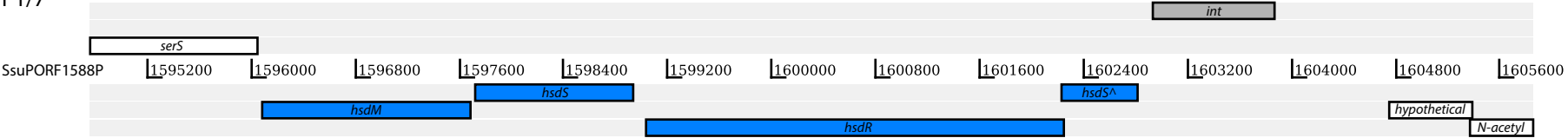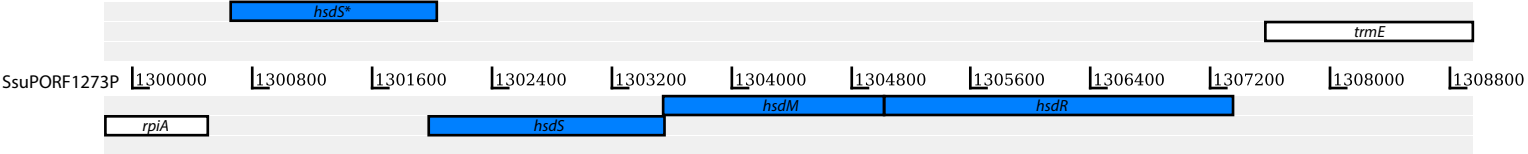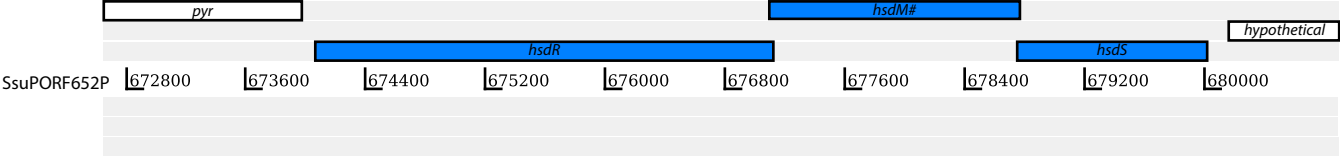

TL13

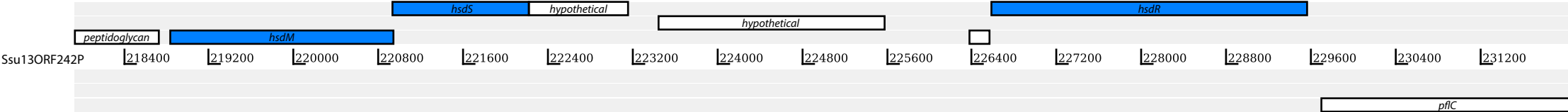

Supplement: Supplementary file 1 [file pathogens-05-00062-s001.zip › figure_s2.pdf]

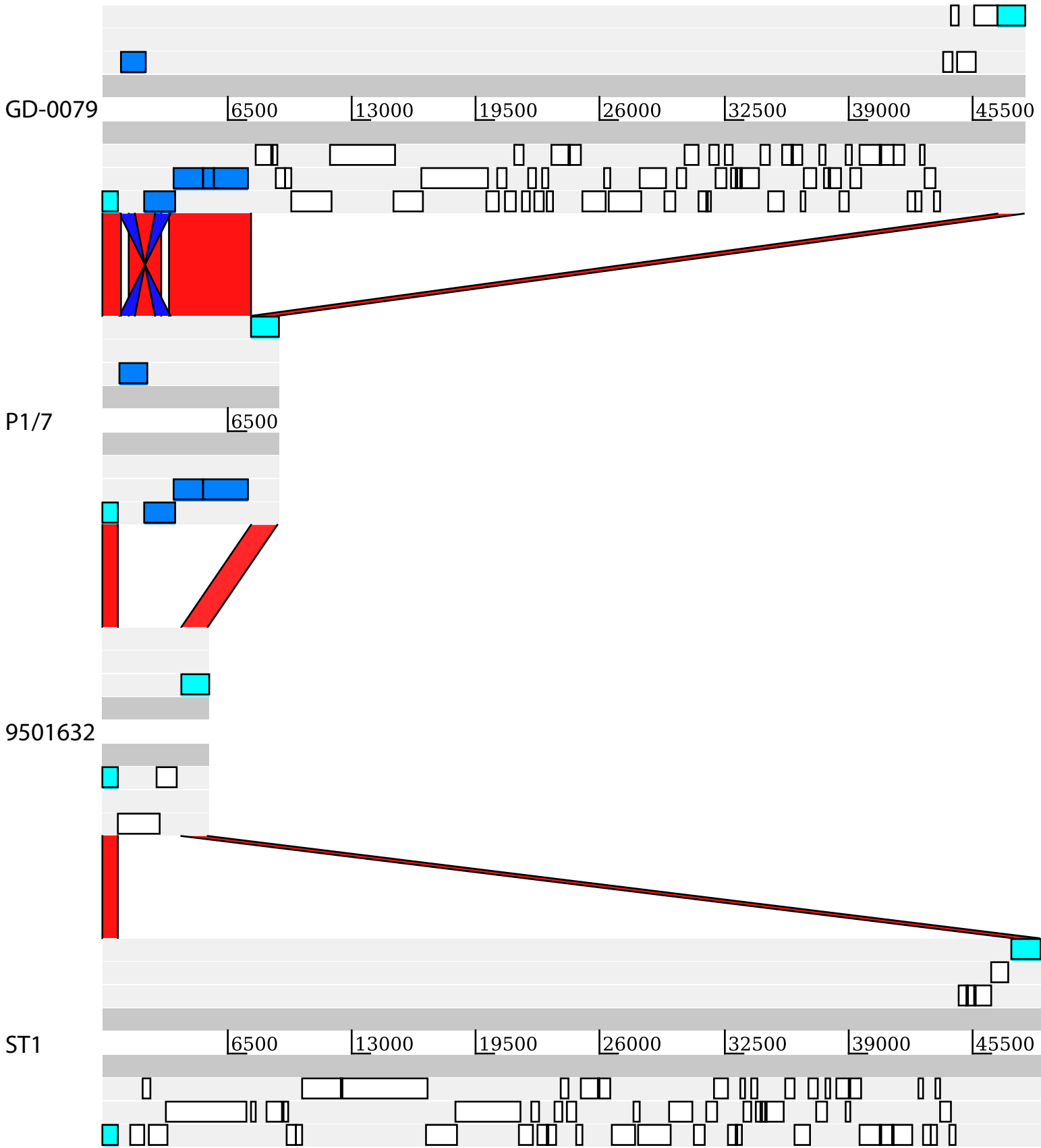

Supplement: Supplementary file 1 [file pathogens-05-00062-s001.zip › figure_s3.pdf]

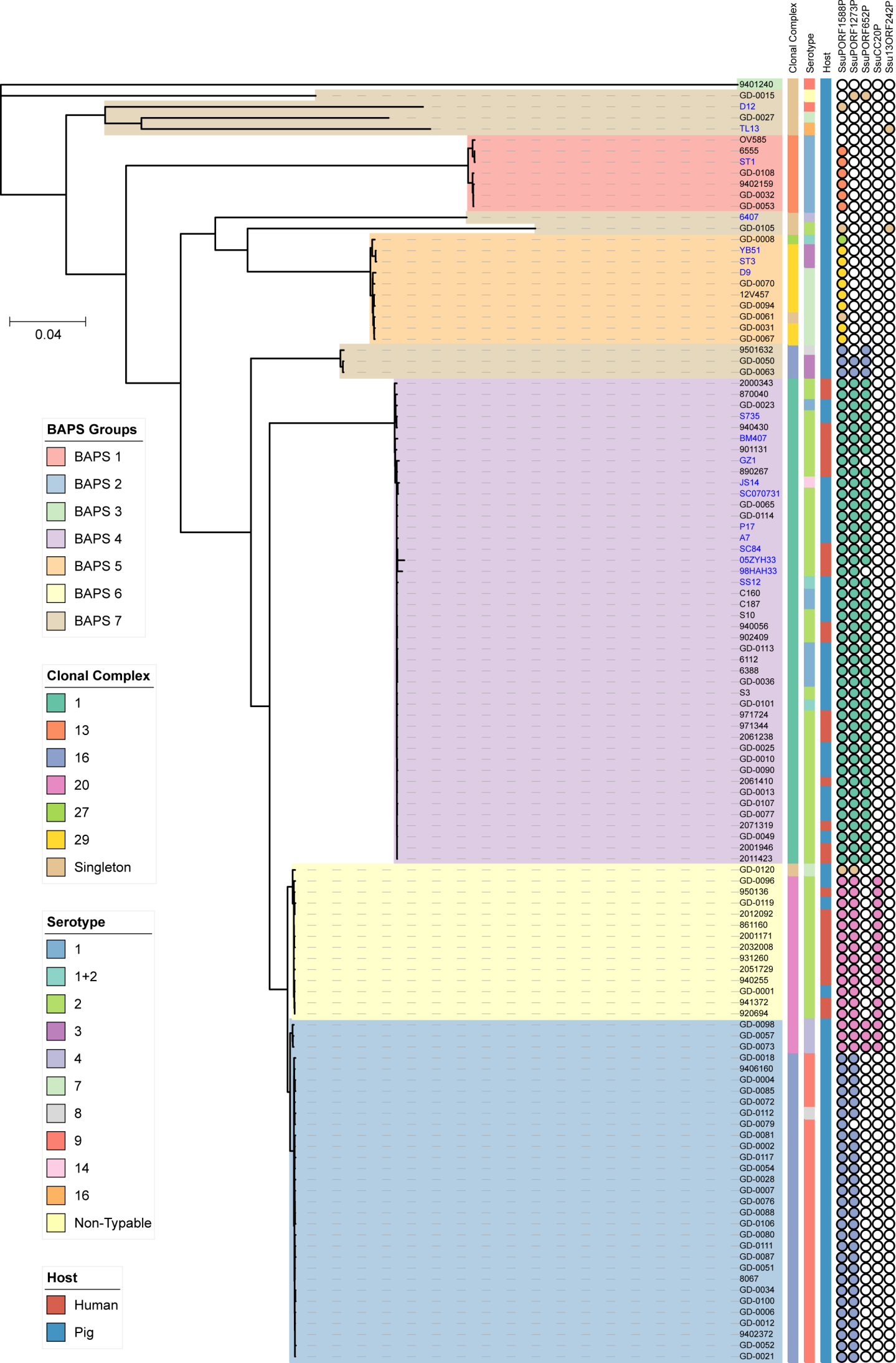

Supplement: Supplementary file 1 [file pathogens-05-00062-s001.zip › figure_s4.pdf]
